# Transcriptomic analysis of asthma and allergic rhinitis reveals CST1 as a biomarker of unified airways

**DOI:** 10.3389/fimmu.2023.1048195

**Published:** 2023-01-17

**Authors:** Mingming Wang, Li Gong, Yang Luo, Shaojuan He, Xianxing Zhang, Xinyu Xie, Xuezhong Li, Xin Feng

**Affiliations:** Department of Otorhinolaryngology, Qilu Hospital of Shandong University, National Health Commission (NHC) Key Laboratory of Otorhinolaryngology, Shandong University, Jinan, Shandong, China

**Keywords:** CST1, unified airways, asthma, allergic rhinitis, transcriptomic analysis

## Abstract

**Background:**

Allergic rhinitis (AR) is an important risk factor for the development of asthma. The “unified airway” theory considers the upper and lower airways as a morphological and functional whole. However, studies exploring biomarkers linking the upper and lower airways in allergic disease are lacking, which may provide insight into the mechanisms underlying AR comorbid asthma.

**Purpose:**

To integrate bioinformatics techniques to explore biomarkers in airway allergic diseases, and to provide a molecular etiology profile for preventing the development of asthma in AR patients.

**Methods:**

Biomarkers were screened by identifying key genes common between AR and asthma through WGCNA and differential gene analysis. GO and KEGG analyses were performed using DAVID. Immuno-infiltration analysis was performed by CIBERSORTx. The predictive value of CST1 to distinguish Th2-high asthma was determined by ROC curves. GSEA was used to analyze the signaling pathways involved in CST1. TargetScan and miRNet were combined with GSE142237 to construct ceRNA network. CMap was used to explore potential therapeutic drugs.

**Results:**

Validation of datasets showed that CST1 was the only gene that was up-regulated in both upper and lower airways in patients with AR and asthma, and correlation heatmaps showed that CST1 was the gene with the highest sum of correlation coefficients. GO and KEGG analysis demonstrated that the lower airways of AR patients were mainly involved in inflammatory and immune responses, similar to asthma. Immune infiltration showed that CST1 was mainly positively correlated with activated CD4 memory T cells. According to the ROC curve, CST1 showed excellent diagnostic efficiency for Th2-high asthma. GSEA indicated that CST1 was involved in the FcϵRI signaling pathway and O-glycan biosynthesis. A ceRNA network including the lncRNAs KCNQ1OT1 and NEAT1 was constructed. Four drugs, including verrucarin-A, had the potential to prevent the development of asthma in AR patients. In addition, corticosteroids were found to downregulate CST1 expression.

**Conclusion:**

CST1 plays a key role in the development of AR comorbid asthma and may be a biomarker for airway allergic diseases. Targeted treatment of CST1 has the potential to prevent the development of asthma in AR patients and deserves further study.

## 1 Introduction

Allergic rhinitis (AR) and asthma are common chronic inflammatory diseases of the upper and lower airways, respectively (1). Epidemiological studies have shown that AR is closely related to asthma (2), 10.5%-43% of AR patients suffer from asthma, and the prevalence of AR in asthma patients is as high as 74%-100% (3, 4). There is clear evidence that AR is an important risk factor for the development of asthma (5–7). AR in childhood is associated with a 2- to 7-fold increased risk of new-onset asthma after childhood (8). Asthma patients with AR are significantly younger at diagnosis of asthma compared to asthma patients without AR (9). Comorbid AR is also a marker of worse asthma control and treatment of comorbid AR can improve asthma symptoms (10). In pathophysiology, asthma-like inflammatory and remodeling processes have been detected in the lower airways of non-asthmatic patients with AR (11, 12). Clinical evidence also shows that patients with pure AR are accompanied by bronchial inflammation characterized by increased fractional exhaled nitric oxide, abnormal small airway function, and bronchial hyperresponsiveness, which is similar to asthma (13, 14). These results indicate that inflammation of the upper airway can lead to abnormal changes in the mucosa of the lower airway, as the concept of “unified airway” proposes: the upper airway and the lower airway are a morphological and functional whole with common barrier and immune-related features (15).

Regarding the mechanism of the effect of the upper airway on the lower airway, although various hypotheses have been proposed, such as allowing more allergens to enter the lower airway and releasing cytokines from the nasal mucosa, the specific mechanism remains poorly understood (16). To date, no studies have combined asthma and AR transcriptomic signatures for analysis, which may provide an opportunity to identify biomarkers of airway allergic diseases and to further understand the concept of “unified airway” from transcriptomics. Therefore, in this study we analyzed upper and lower airway transcriptome data collected from public databases of patients suffering from AR and/or asthma by applying a bioinformatics approach to explore biomarkers potentially linking the upper and lower airways, constructing immune cell infiltration and competitive endogenous RNA (ceRNA) networks to further reveal their molecular mechanisms.

This study showed that CST1 and its related molecular functions played a key role in AR comorbid with asthma. CST1 is the coding gene of cystatin SN, which is a secreted protein that functions as a cysteine protease inhibitor in the extracellular matrix (17). CST1 has been found to be upregulated in various airway inflammatory diseases (18–20). Here we further demonstrated its key role in the development of AR comorbid asthma and its potential to be a biomarker for airway allergic diseases, especially for Th2-type asthma.

## 2 Materials and methods

### 2.1 Microarray dataset download

The microarray datasets GSE19187, GSE67472, GSE41861, GSE89809, GSE142237 and GSE101720 on asthma and AR were downloaded from the Gene Expression Omnibus (GEO) database(https://www.ncbi.nlm.nih.gov/geo/) established by the national center for biotechnology information (21). GSE19187 contains gene expression profiles of nasal epithelial cells from 14 children with AR, 6 children with AR and concomitant uncontrolled asthma, 7 children with AR and concomitant controlled asthma, and 11 healthy controls. GSE67472 contains gene expression profile data from bronchial epithelial brushes obtained from 62 subjects with mild to moderate asthma (Th2-high = 40, Th2-low = 22) and 43 control subjects without asthma. GSE41861 contains gene expression profiling data from nasal and bronchial epithelial brushes from 54 asthma patients and 30 healthy control subjects. GSE101720 contains gene expression profiling data from nasal and bronchial epithelial brushes of 7 asthma comorbid AR, 10 AR, and 9 healthy subjects. GSE89809 contains bronchial epithelial gene expression profile data from 38 asthma patients, 13 of whom were not on inhaled corticosteroids (ICS) medication, and the remaining 25 patients inhaled different doses of corticosteroids for asthma. GSE142237 contains bronchial epithelial microRNA (miRNA) expression profiles obtained by bronchoscopy from 8 asthma patients and 4 healthy controls. All datasets are in compliance with GEO’s upload regulations, and have been background corrected and normalized. Details about each dataset are shown in **Table 1**. (Detailed information on the inclusion criteria and diagnostic criteria for each dataset is provided in **Supplementary Table S1**). All data in this study came from online public databases and adhered to the corresponding ethical standards.

**Table 1:** The details of GEO datasets for the research.

GSE	PMID	Sample size (n)	Organization type	Platform
GSE19187	22005912	Allergic rhinitis: 14	14 nasal epithelial cells	GPL6244
		AR with UA: 6	6 nasal epithelial cells	
		AR with CA: 7	7 nasal epithelial cells	
		Control: 11	11 nasal epithelial cells	
GSE67472	25611785	Asthma: 62	62 bronchial epithelial cells	GPL16311
		Th2-high: 40		
		Th2-low: 22		
		Control: 43	43 bronchial epithelial cells	
GSE101720	30190271	AR and asthma: 7	7 nasal epithelial cells	GPL18573
			7 bronchial epithelial cells	
		Allergic rhinitis: 10	10 nasal epithelial cells	
			10 bronchial epithelial cells	
		Control: 9	9 nasal epithelial cells	
			9 bronchial epithelial cells	
GSE41861	NA	Asthma: 54	40 nasal epithelial cells	GPL570
			51 bronchial epithelial cells	
		Control: 30	17 nasal epithelial cells	
			30 bronchial epithelial cells	
GSE89809	28933920	Asthma: 38		GPL13158
		Use of ICS: 25	25 bronchial epithelial cells	
		No ICS: 13	13 bronchial epithelial cells	
GSE142237	33945508	Asthma: 8	8 bronchial epithelial cells	GPL18058
(microRNA)		Control: 4	4 bronchial epithelial cells	

GSE, GEO Series; PMID, PubMed Unique Identifier; AR, Allergic rhinitis; UA, Uncontrolled Asthma; CA, Controlled Asthma; ICS, Inhaled Corticosteroids.

### 2.2 Construction of a weighted gene co-expression network to identify key genes co-associated with asthma and AR

Weighted gene co-expression network analysis (WGCNA) identifies potential gene interactions and correlations with phenotypes by identifying gene co-expression relationships in samples, and is used to explore the complex relationships between gene expression profiles and phenotypes (22). The WGCNA package (version 1.71) of R software was used to analyze co-expressed genes in the GSE19187 and GSE67472 to construct a weighted gene co-expression network. By calculating the standard deviation of each gene, the top 25% of genes were ranked in descending order by standard deviation value and used to construct a WGCNA network for subsequent analysis. The scale-free network of gene expression profiles was constructed based on the correlation coefficient R^2^ >0.90 using the pick soft threshold function. Finally, the dynamic tree cut algorithm was used to identify the modules, and 30 was selected as the minimum number of genes for each module. The correlation between different modules and diseases was calculated separately to assess the association between modules and diseases, and the heatmap of module-disease correlation was drawn by R software. The module with the highest correlation was used as the key module, and the genes with gene significance (GS) > 0.5 and P-value GS < 0.05 in the key module had strong correlation with both diseases and modules. These genes were used as candidate genes for subsequent analysis.

Use R software to convert probe names in the microarray to official gene symbols. For a gene with multiple probes, the average value of the probes was taken as the expression value of the gene for subsequent analysis. Subsequently, differential gene analysis was performed using the Limma R package (version 3.48.3) applying the empirical Bayes method for GSE19187 and GSE67472 to compare gene expression profile data from asthma/AR patients with healthy controls to identify differentially expressed genes (DEGs) for asthma and AR (23). The criteria for screening disease-related DEGs were |log2 FC| > 1 and *p*-value < 0.05. Candidate genes with differential expression in key modules associated with AR and asthma were identified as key genes co-associated with asthma and AR by the tool Venn diagrams (https://bioinformatics.psb.ugent.be/webtools/Venn/).

### 2.3 GO and KEGG analysis and validation of key genes

Differential gene analysis was performed on the gene expression matrix of GSE101720 and GSE41861 to explore the DEGs in the upper and lower airways of AR/asthma patients. Subsequently, gene ontology (GO) analysis and Kyoto encyclopedia of gene and genomes (KEGG) pathway enrichment analysis were performed on DEGs screened from the bronchial epithelium of AR patients and nasal epithelium of asthma patients using DAVID online software(https://david.ncifcrf.gov/) to explore the biological functions and related pathways of DEGs (24). GO analysis and KEGG enrichment analysis were statistically significant at *P* < 0.05. GO analysis included three components, biological process (BP), cellular component (CC), and molecular function (MF). The DEGs of GSE101720 and GSE41861 were also used to verify the expression levels of key genes in AR and asthma. Interactions between key gene-encoded proteins were then explored through the STRING database(https://cn.string-db.org/) (25). By using the Venn Diagram tool to identify key genes that are jointly up- and down-regulated in the upper and lower airways of asthma and AR patients, these genes may serve as the biomarker for allergic disease in the upper and lower airways.

### 2.4 Correlation of biomarker expression in the upper and lower airways

To investigate the relationship between biomarker expression levels in the upper and lower airways, Pearson correlation analysis was performed on the expression data of CST1 (Cystatin SN) gene in the upper and lower airways of AR and asthma patients. Pearson correlation analysis was also performed between CST1 and the remaining key genes in the lower airways of AR patients and the upper airways of asthma patients.

### 2.5 Differences in CST1 expression levels between different comorbidities

Statistical analysis of CST1 gene expression in the upper and lower airways of asthma comorbid AR, AR, and healthy subjects in GSE101720 was performed applying the Mann-Whitney U test. GSE19187 was used to analyze the expression levels of CST1 in AR, AR with uncontrolled asthma, AR with controlled asthma and healthy subjects to explore the expression level of CST1 in different comorbidities.

### 2.6 Immune infiltration analysis

CIBERSORTx is an analytical tool for assessing the abundance of immune cell subpopulations in tissue samples by applying a deconvolution algorithm using gene expression data (26). GSE41861 gene expression profile data from asthmatic and healthy subjects were uploaded to CIBERSORTx to analyze the abundance of immune cell subpopulations in the bronchial epithelium of asthmatic patients. LM22 Signature matrix file, which contains 22 immune cell components, was used as a reference for cell quantification. Pearson correlation analysis was used for correlation analysis between immune cells and genes.

### 2.7 Analysis of the correlation between CST1 and Th2-high asthma

Asthmatic subjects in the GSE41861 microarray dataset were divided into Th2-high and Th2-low asthma groups based on the expression of three signature genes of type 2 inflammation, SERPINB2, POSTN and CLCA1, applying Euclidean distance clustering analysis (27, 28). The predictive value of CST1 to distinguish Th2-high asthma was determined using receiver operating characteristic (ROC) curves. GSE67472 containing 40 patients with Th2-high asthma and 22 patients with Th2-low asthma was then applied to validate the efficiency of CST1 in predicting Th2-high asthma. In addition, the correlation of CST1 with IL-13 and the signature gene CLCA1 of type 2 inflammatory in the bronchial epithelium of asthmatic patients was analyzed. GSE101720 microarray dataset were used to analyze the correlation of CST1 with CLCA1 in the upper airway.

### 2.8 Gene set enrichment analysis

Gene set enrichment analysis (GSEA) analyzed the molecular signaling pathways of CST1 involved in asthma at the gene set level by assessing the distribution trends of genes in a pre-defined gene set in phenotype-related microarray data to determine their contribution to phenotype (29). The gene expression profiles of the samples in GSE41861 were divided into two groups, CST1 high expression and CST1 low expression, based on the expression of CST1 with a median cut-off value. All detected genes were further analyzed using GSEA software (V4.2.3) to explore the molecular signaling pathways involved in CST1 from the perspective of gene sets. The “c2.cp.kegg.v7.5.1.symbols” downloaded from the GSEA molecular signature database was selected as the reference gene set to perform GSEA analysis. Significantly enriched pathways were screened based on *P*-value < 0.05 and normalized enrichment score (NES) ranking.

### 2.9 Exploration ceRNA network of the key genes

In recent years, it has been found that non-coding RNAs, including long non-coding RNA (lncRNA) and miRNA, do not encode proteins or encode only small peptides, but still play important roles in physiological regulation and pathogenesis (30, 31). miRNAs cause gene silencing by binding mRNAs, and lncRNAs inhibit the function of miRNAs by competing with them, this regulatory mechanism called ceRNA regulation (32). Differential analysis of the GSE142237 expression matrix was performed using the Limma R package to obtain differentially expressed miRNAs. Potential miRNAs of 4 key genes were retrieved by TargetScan database(https://www.targetscan.org/vert_80/). According to the ceRNA network mechanism, miRNAs should be opposite to mRNA expression trends, and possible miRNAs were identified by the tool Venn diagrams. The miRNet database (https://www.mirnet.ca/) was used to predict the lncRNAs involved in regulation, and lncRNAs involved in the regulation of multiple miRNAs were used to construct the ceRNA network.

### 2.10 Small molecule drug candidates for comorbidity prevention

The Connectivity Map (CMap) database performs transcriptome-level sequencing of cell lines treated with different types of reagents and analyzes the differential gene profiles caused by these reagents relative to normal cell lines (33). DEGs and their expression trends in the lower airways of AR patients were imported and matched to differential gene profiles in the CMap database. Compounds with negative enrichment scores had the potential to reverse the expression trends of uploaded gene expression. Subsequently, the top 3 small molecule compounds capable of reversing the expression of uploaded genes were screened by enrichment score ranking. These compounds have the potential to prevent the development of asthma in AR patients. Watson et al. demonstrated that intranasal corticosteroids treatment of upper airway inflammation may indirectly improve asthma symptoms and reduce bronchial hyperresponsiveness (34). To confirm whether CST1 is a potential therapeutic target for corticosteroids, the effect of corticosteroids on the expression level of bronchial epithelial CST1 in asthma patients was explored by analyzing GSE89809.

## 3 Results

### 3.1 Key genes commonly associated with asthma and AR

**Figure 1:**
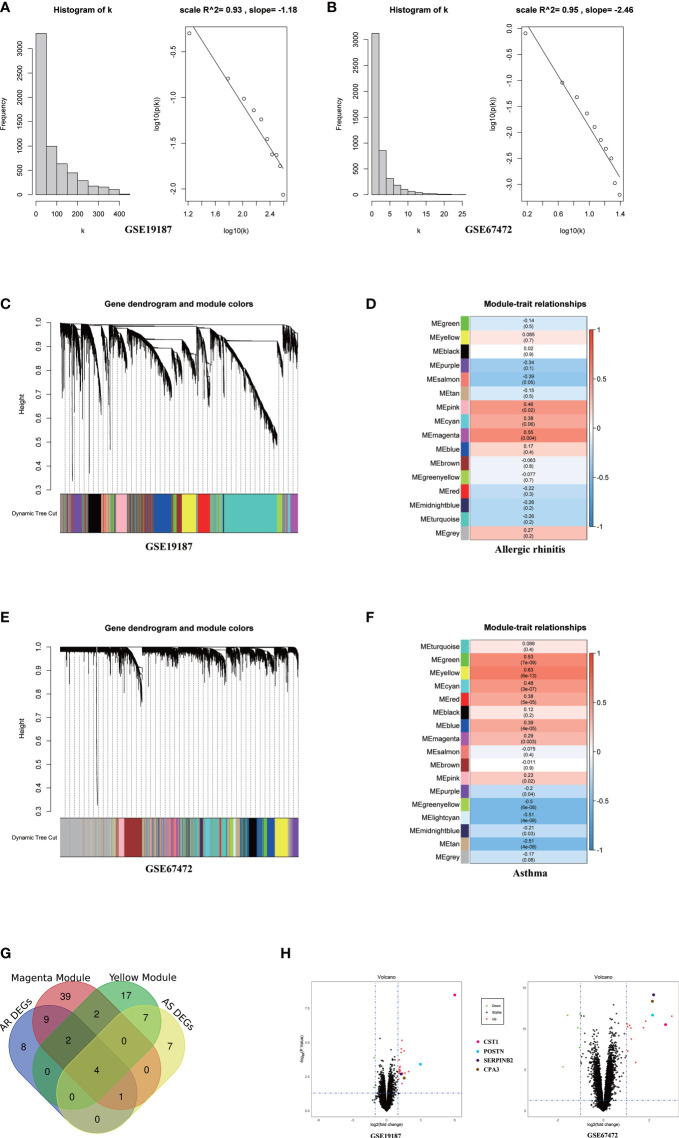
WGCNA analysis of GSE19187 and GSE67472. **(A)** Validation of scale-free networks for GSE19187. The histogram shows the relationship between connectivity (k) and frequency. The scatterplot x-axis represents the log of the overall network connectivity(k), and the y-axis represents the log of the corresponding frequency distribution p(k). There is a negative correlation between k and p(k), and the constructed network conforms to the scale-free network distribution. **(B)** Validation of scale-free networks for GSE67472. **(C)** Clustering dendrograms of genes for GSE19187. Different colors below indicate different co-expression modules. **(D)** The module-trait relationships for GSE19187. Each cell contains the corresponding correlation and p-value; red for positive correlation and blue for negative correlation. **(E)** Clustering dendrograms of genes for GSE67472. Different colors below indicate different co-expression modules. **(F)** The module-trait relationships for GSE67472. **(G)** The Venn diagram shows that a total of 4 key genes were screened from the DEGs and network key modules. **(H)** The volcano plot shows that four key genes are significantly up-regulated in both GSE19187 and GSE67472. DEGs, differentially expressed genes; AR, Allergic Rhinitis; AS, Asthma.

The top 6134/4744 (top 25%) genes in descending order of standard deviation values in GSE19187 and GSE67472 were used to construct co-expression modules using the WGCNA algorithm. To ensure that the network conforms to the scale-free network, we performed network topology analysis and constructed the network with the correlation coefficient R^2^ >0.90. The correlation coefficient R^2^ and the average connectivity of the scale-free network both reached a steady state. There was a power-law distribution of connectivity in the scale-free network, connectivity (k) was negatively correlated with p-(k) in the network, and the constructed network conformed to the scale-free network distribution (**Figures 1A; B**). Fifteen modules were identified in GSE19187, ranging in size from 1720 genes (Turquoise) to 67 genes (Midnight blue) (**Figure 1C**), and the relationship between modules and clinical traits was calculated (**Supplementary Table S2**), in addition, a gray module was used to collect genes not assigned to any modules and was excluded from further analyses. The magenta module was closely correlated with AR (r = 0.55, *P* = 0.004) (**Figure 1D**), and there were 57 candidate genes in the magenta module that strongly related to both the disease and the module. Sixteen modules were identified in GSE67472 (Gray module was excluded), ranging in size from 593 genes (Turquoise) to 74 genes (Light cyan) (**Figure 1E**), with the yellow module closely correlated with asthma (r = 0.63, *P* = 6e-13) (**Figure 1F**), and there were 32 candidate genes which were strongly correlated with both disease and module in the yellow module (**Supplementary Table S3**).

Through differentially expressed genes analysis, 24/19 DEGs were filtered from GSE19187 and GSE67472 respectively. Venn diagram analysis results demonstrated that CST1, POSTN, CPA3, and SERPINB2 were not only candidate genes in key modules but also DEGs in AR and asthma. (**Figure 1G**; **Table 2**). In addition, the volcano plot showed that CST1, POSTN, CPA3, and SERPINB2 were significantly up-regulated in the bronchial epithelium of asthma and nasal epithelium of AR (**Figure 1H**; **Table 3**). CST1, POSTN, CPA3, and SERPINB2 were key genes that potentially linked AR and asthma.

**Table 2:** Correlations between 4 key genes and diseases and modules.

Module	Gene	Disease	GS	P Value-GS	MM	P Value-MM
Magenta	CST1	Allergic rhinitis	0.85	6.1E-08	0.77	6.12E-06
Magenta	CPA3	Allergic rhinitis	0.52	8.4E-03	0.77	5.52E-06
Magenta	POSTN	Allergic rhinitis	0.61	1.2E-03	0.84	1.31E-07
Magenta	SERPINB2	Allergic rhinitis	0.54	4.9E-03	0.71	5.95E-05
Yellow	CST1	Asthma	0.58	7.58E-11	0.84	1.19E-29
Yellow	CPA3	Asthma	0.64	1.41E-13	0.78	2.90E-22
Yellow	POSTN	Asthma	0.61	6.01E-12	0.78	4.46E-23
Yellow	SERPINB2	Asthma	0.66	2.50E-14	0.86	2.95E-31

GS, Gene significance; MM, module membership.

**Table 3:** Differential analysis results of 4 key genes screened by WGCNA network.

Gene		GSE19187		GSE67472	
Symbol	Description	logFC	P Value	logFC	P Value
CST1	Cystatin SN	6.00	3.3E-09	2.71	3.00E-11
CPA3	Carboxypeptidase A3	2.98	3.9E-04	2.15	2.16E-12
POSTN	Periostin	1.37	2.2E-03	2.19	7.19E-15
SERPINB2	Serpin Family B Member 2	1.55	4.1E-03	2.14	4.36E-14

### 3.2 GO and KEGG analysis

**Figure 2:**
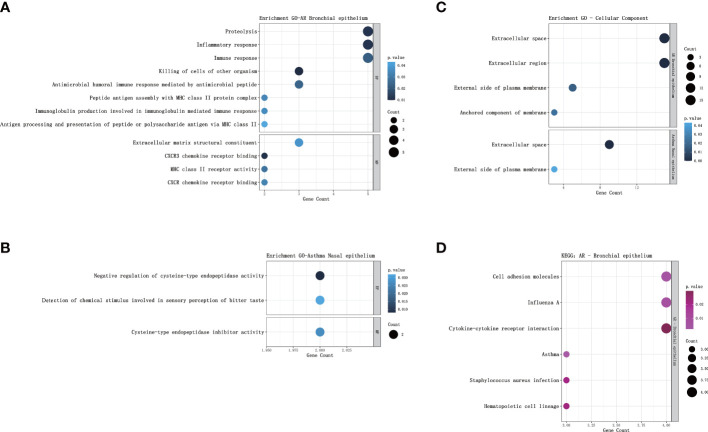
GO and KEGG pathway enrichment analysis of DEGs. **(A)** Bubble chart illustrating the terminology of significant enrichment of bronchial epithelial DEGs in AR patients in terms of BP and MF. The horizontal axis represents the gene count, and the vertical axis represents the GO terminology. The size of the dots represents the number of enriched genes, and the shades of color represent *P*-values. **(B)** Bubble chart showing the terminology of significant enrichment of nasal epithelial DEGs in asthma patients in terms of BP and MF. **(C)** Bubble chart showing the terminology of significant enrichment of DEGs in the nasal epithelium of asthma patients and bronchial epithelium of AR patients in terms of CC. **(D)** Bubble chart showing enriched signaling pathways associated with bronchial epithelial DEGs in AR patients. GO, gene ontology; KEGG, Kyoto Encyclopedia of Genes and Genomes; DEGs, differentially expressed genes; BP, Biological Processes; MF, Molecular Function; AR, Allergic Rhinitis.

GO analysis of bronchial epithelial DEGs in AR patients showed that DEGs were mainly enriched in BP of the inflammatory and immune response. MF terms enriched for DEGs were CXCR3 chemokine receptor binding and CXCR chemokine receptor binding (**Figure 2A**; **Table 4**). GO analysis of nasal epithelial DEGs in asthma patients demonstrated that DEGs were mainly enriched in the BP of negative regulation of cysteine-type endopeptidase activity. MF terms enriched for DEGs were cysteine-type endopeptidase inhibitor activity (**Figure 2B**). In terms of CC, DEGs in the bronchial epithelium of AR patients and the nasal epithelium of asthma patients were mainly distributed in the extracellular space and external side of the plasma membrane (**Figure 2C**).

**Table 4:** Results from GO enrichment analysis of DEGs.

Category	Term	Description	Gene count	P value	Gene ID
Allergic rhinitis-bronchial epithelia					
BP	GO:0006508	proteolysis	5	0.012	TMPRSS11A/CPA3/TPSB2/PAPPA/TPSAB1
BP	GO:0006954	inflammatory response	5	0.012	SELP/CXCL11/CXCL9/NLRP2/MS4A2
BP	GO:0006955	immune response	5	0.022	IL1RL1/CSF3/MS4A2/HLA-DQA2/HLA-DQB1
BP	GO:0031640	killing of cells of other organism	3	0.010	CXCL11/CXCL9/GNLY
BP	GO:0061844	antimicrobial humoral immune response mediated by antimicrobial peptide	3	0.024	CXCL11/CXCL9/GNLY
BP	GO:0002503	peptide antigen assembly with MHC class II protein complex	2	0.034	HLA-DQA2/HLA-DQB1
BP	GO:0002381	immunoglobulin production involved in immunoglobulin mediated immune response	2	0.036	HLA-DQA2/HLA-DQB1
BP	GO:0002504	antigen processing and presentation of peptide or polysaccharide antigen *via* MHC class II	2	0.043	HLA-DQA2/HLA-DQB1
CC	GO:0005615	extracellular space	15	0.001	CPA3/CSF3/POSTN/TPSB2/CXCL9/MUC5AC/SELP/CXCL11/CST1/CILP/GNLY/PAPPA/FETUB/TFF1/TPSAB1
CC	GO:0005576	extracellular region	15	0.001	CPA3/TMPRSS11A/CSF3/CXCL9/PSCA/LYPD2/MUC5AC/IL1RL1/CXCL11/GNLY/PAPPA/CEACAM5/FETUB/TFF1/TPSAB1
CC	GO:0009897	external side of plasma membrane	5	0.016	SELP/IL1RL1/CXCL9/MS4A2/HLA-DQB1
CC	GO:0031225	anchored component of membrane	3	0.024	PSCA/CEACAM5/LYPD2
MF	GO:0005201	extracellular matrix structural constituent	3	0.038	POSTN/CILP/MUC5AC
MF	GO:0048248	CXCR3 chemokine receptor binding	2	0.011	CXCL11/CXCL9
MF	GO:0032395	MHC class II receptor activity	2	0.029	HLA-DQA2/HLA-DQB1
MF	GO:0045236	CXCR chemokine receptor binding	2	0.033	CXCL11/CXCL9
Asthma-nasal epithelia					
BP	GO:2000117	negative regulation of cysteine-type endopeptidase activity	2	0.008	CST1/CST4
BP	GO:0001580	detection of chemical stimulus involved in sensory perception of bitter taste	2	0.032	CST1/CST4
MF	GO:0004869	cysteine-type endopeptidase inhibitor activity	2	0.027	CST1/CST4
CC	GO:0005615	extracellular space	9	0.001	SERPINB10/CPA3/POSTN/CST1/MUC2/ITLN1/CLCA1/CST4/CCL26
CC	GO:0009897	external side of plasma membrane	3	0.043	CD200R1/CSF2RB/MS4A2

KEGG analysis showed that the bronchial epithelial DEGs of AR patients were mainly involved in the signaling pathways of asthma, cell adhesion molecules, influenza A, staphylococcus aureus infection, hematopoietic cell lineage, and cytokine-cytokine receptor interaction (**Figure 2D**; **Table 5**). Due to the low number of DEGs in the nasal epithelium of asthmatic patients, it failed to enrich significant pathways in the KEGG analysis.

**Table 5:** KEGG pathways analysis results of DEGs.

Pathway ID	Description	Count	P-value	Genes
Allergic rhinitis-bronchial epithelia				
hsa05310	Asthma	3	0.002	MS4A2, HLA-DQA2, HLA-DQB1
hsa04514	Cell adhesion molecules	4	0.005	SELP, NLGN4Y, HLA-DQA2, HLA-DQB1
hsa05164	Influenza A	4	0.007	TPSB2, TPSAB1, HLA-DQA2, HLA-DQB1
hsa05150	Staphylococcus aureus infection	3	0.021	SELP, HLA-DQA2, HLA-DQB1
hsa04640	Hematopoietic cell lineage	3	0.022	CSF3, HLA-DQA2, HLA-DQB1
hsa04060	Cytokine-cytokine receptor interaction	4	0.030	IL1RL1, CSF3, CXCL11, CXCL9
Asthma-nasal epithelia				
NA				

### 3.3 Validation of key genes indicated CST1 was a biomarker of upper and lower airways

**Figure 3:**
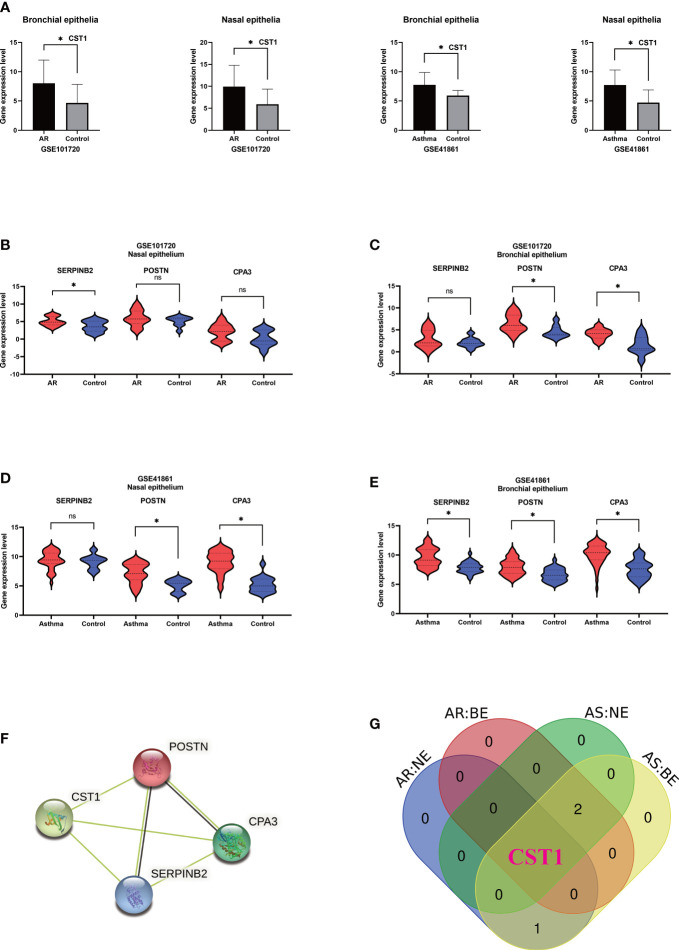
Validation of key genes and identification of upper and lower airway biomarkers. **(A)** Expression levels of CST1 in nasal and bronchial epithelium of allergic rhinitis and asthma patients in GSE101720 and GSE41861. **(B)** Expression levels of three key genes in the nasal epithelium of patients with allergic rhinitis in GSE101720. **(C)** Expression levels of three key genes in the bronchial epithelium of allergic rhinitis patients in GSE101720. **(D)** Expression levels of three key genes in the nasal epithelium of asthma patients in GSE41861. **(E)** Expression levels of three key genes in the bronchial epithelium of asthma patients in GSE41861. **(F)** Interactions between proteins encoded by four key genes. **(G)** Venn Diagram shows that CST1 is the only key gene whose expression level is up-regulated in nasal epithelium and bronchial epithelium of both AR and asthma patients. AR, Allergic Rhinitis; AS, Asthma; NE, Nasal Epithelium; BE, Bronchial Epithelium; ns, no significance; *P < 0.05.

Analysis of GSE41861 and GSE101720 showed that (details of DEGs of GSE41861 and GSE101720 in **Supplementary Table S4**) the expression level of CST1 was significantly up-regulated in both nasal and bronchial epithelium of AR and asthma patients (**Figure 3A**). In addition, the expression level of SERPINB2 was up-regulated in the nasal epithelium of AR patients (**Figure 3B**), the expressions of POSTN and CPA3 were up-regulated in the bronchial epithelium of AR patients (**Figure 3C**), the expressions of POSTN and CPA3 were up-regulated in the nasal epithelium of asthma patients (**Figure 3D**), and the expressions of SERPINB2, POSTN, and CPA3 were up-regulated in the bronchial epithelium of asthma patient (**Figure 3E**). The STRING database showed that CST1, POSTN, CPA3, and SERPINB2 proteins had a certain interaction (minimum required interaction score: 0.15) (**Figure 3F**). Venn Diagram tool analysis showed that CST1 was the only key gene that was up-regulated in both nasal epithelium and bronchial epithelium of AR and asthma patients (**Figure 3G**). These results indicated that CST1 had potential as a unified airway biomarker.

### 3.4 The expression levels of CST1 in the upper and lower airways were positively correlated

**Figure 4:**
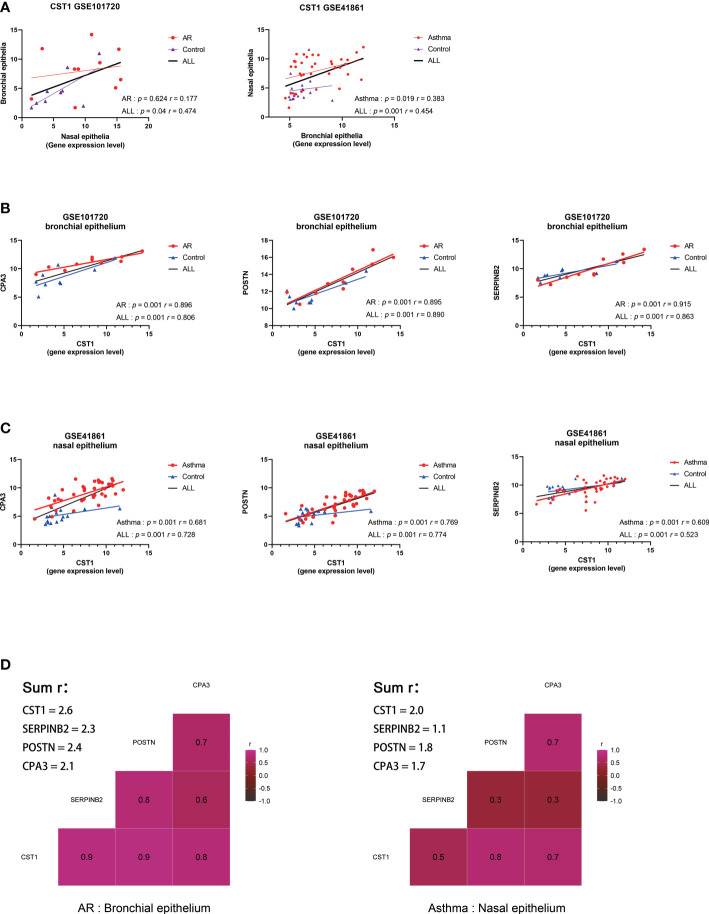
Correlation analysis of key genes. **(A)** Correlation analysis and scatterplot of CST1 expression levels in nasal and bronchial epithelium in GSE101720 and GSE41861. **(B)** Correlation analysis and scatter plot of the expression levels of CST1 and key genes CPA3, POSTN, and SERPINB2 in the bronchial epithelium of AR patients. **(C)** Correlation analysis and scatter plot of the expression levels of CST1 and key genes CPA3, POSTN, and SERPINB2 in the nasal epithelium of asthma patients. **(D)** Heatmap of the correlation between CST1 and key genes CPA3, POSTN, and SERPINB2 in the bronchial epithelium of AR patients and nasal epithelium of asthma patients. AR, Allergic Rhinitis.

Correlation analysis on GSE101720 and GSE41861 showed that the expression level of CST1 in the nasal epithelium was positively correlated with that in the bronchial epithelium (**Figure 4A**). Moreover, the expression level of CST1 was significantly positively correlated with the expression levels of POSTN, CPA3, and SERPINB2 in the bronchial epithelium of AR patients (**Figure 4B**) and in the nasal epithelium of asthma patients (**Figure 4C**). The correlation heatmap showed that CST1 was the gene with the highest sum of correlation coefficients, whether in the bronchial epithelium of AR patients or the nasal epithelium of asthmatic patients (**Figure 4D**).

### 3.5 Expression levels of CST1 in different comorbidities

**Figure 5:**
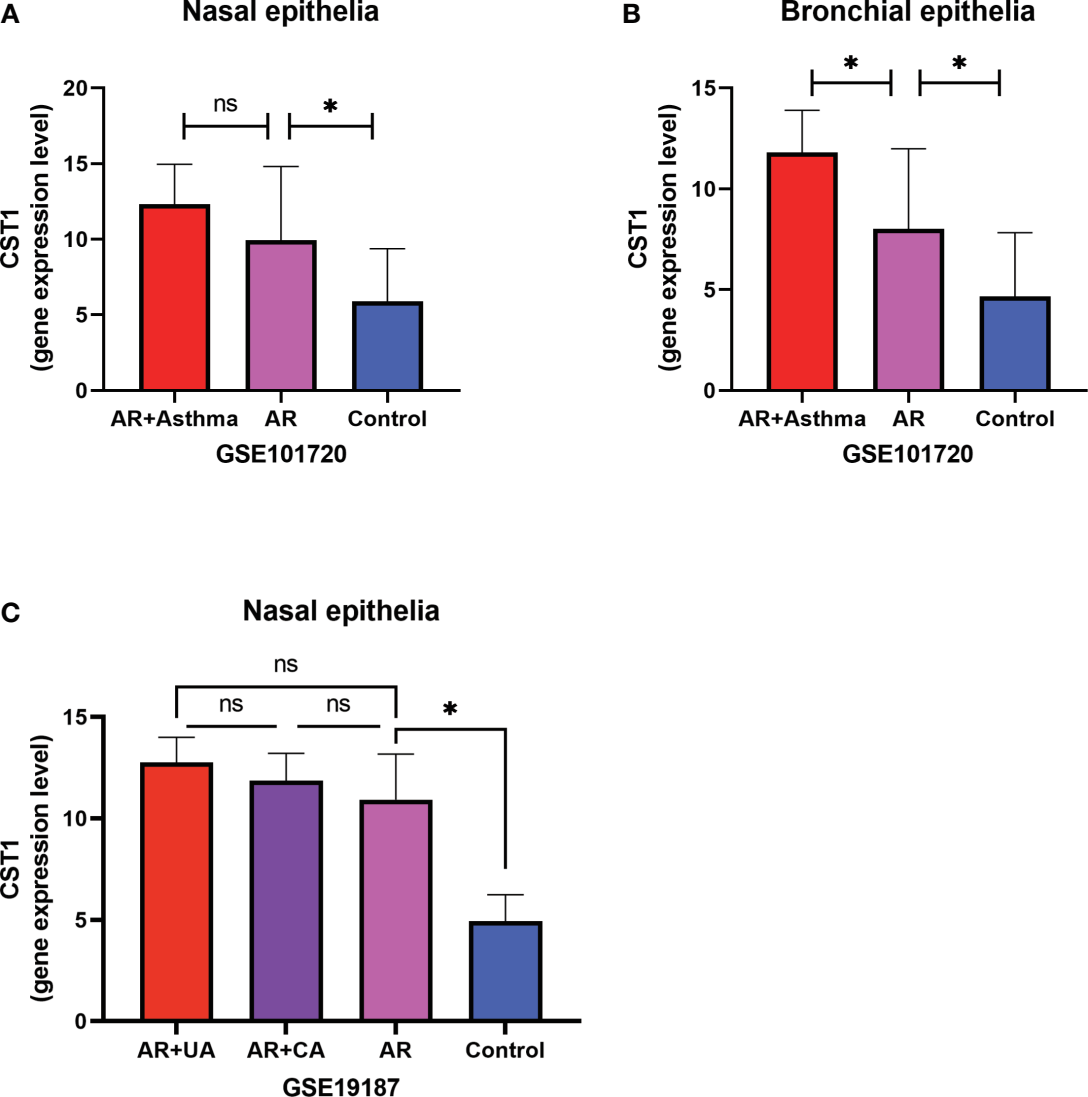
Expression levels of CST1 in different comorbidities. **(A)** Histograms show the expression levels of CST1 in nasal epithelium in AR comorbid asthma, AR alone, and controls in GSE101720. **(B)** Histograms show the expression levels of CST1 in bronchial epithelium in AR comorbid asthma, AR alone and controls in GSE101720. **(C)** The histogram shows the expression levels of CST1 in nasal epithelium in AR comorbid UA, AR comorbid CA, AR alone and control group in GSE19187. AR, Allergic Rhinitis; UA, Uncontrolled Asthma; CA, Controlled Asthma; * P<0.05. ns, no significance.

We performed differential analysis on the expression levels of CST1 in the nasal epithelium and bronchial epithelium among AR comorbid asthma, AR, and healthy subjects in GSE101720 dataset (**Figures 5A; B**). Compared with normal controls, the expression level of CST1 was up-regulated in both nasal and bronchial epithelium of AR comorbid asthma and AR alone. The level of CST1 in the bronchial epithelium of AR comorbid asthma was significantly increased compared to AR alone, while no significant difference was observed in the nasal epithelium between AR comorbid asthma and AR alone, which was further confirmed by GSE19187 dataset, in which AR alone and AR comorbid asthma (controlled or not controlled) presented similar CST1 expression level in the nasal epithelium (**Figure 5C**).

### 3.6 Immune infiltration analysis indicated that CST1 was associated with immune cells

**Figure 6:**
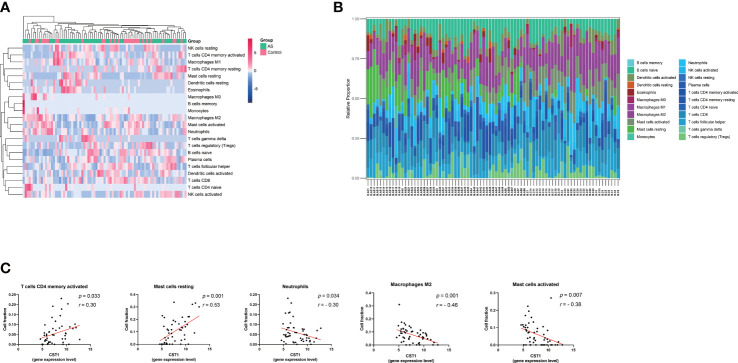
Immune infiltration analysis. **(A)** Clustering heatmap of 22 immune cells. The bar legend in the upper right corner represents the relative fold change in cell abundance, with red for increases and green for decreases. Bar legends above represents sample clusters, with AS in green and controls in pink. **(B)** Histograms show the relative proportion of each immune cell in each sample. Each column represents a sample, and each color represents a cell type. **(C)** Correlation analysis and scatter plot of CST1 expression level and 5 immune cell fractions in the bronchial epithelium of asthma patients. AS, Asthma.

The cluster heatmap about 22-type immune cells showed no significant differences in immune cell composition between asthmatic patients and control subjects (**Figure 6A**), as previous research reported that the proportions of airway macrophages and lymphocytes were basically consistent between healthy and severe disease (35). The histogram showed the proportion of each immune cell in each sample (**Figure 6B**). The relationship between immune cell abundance and CST1 expression levels was analyzed, which showed that CST1 was positively correlated with activated CD4 memory T cells and resting mast cells, and negatively correlated with neutrophils, M2 macrophages, and activated mast cells (**Figure 6C**).

### 3.7 Correlation between CST1 and Th2-high asthma

**Figure 7:**
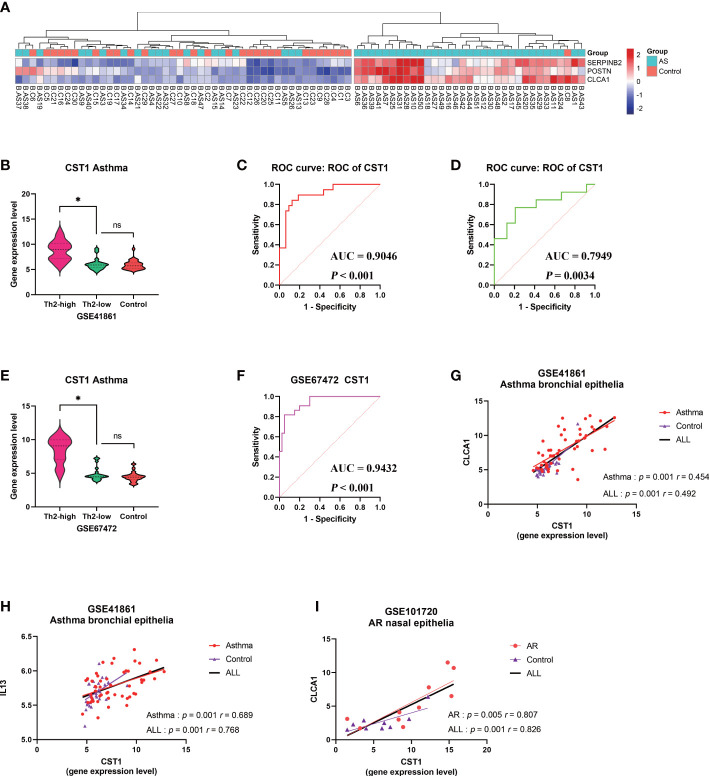
Correlation analysis between CST1 and Th2-high asthma. **(A)** Heatmap showing unsupervised hierarchical clustering of POSTN, CLCA1, and SERPINB2 expression levels in bronchial epithelium. Red represents a high expression level, and blue represents a low expression level. **(B)** Violin plots show the expression levels of CST1 in Th2-high asthma, Th2-low asthma, and controls in GSE41861. **(C)** ROC curves of CST1 expression levels in bronchial epithelium discriminating Th2-high asthma and Th2-low asthma in GSE41861. **(D)** ROC curves of CST1 expression levels in nasal epithelium discriminating Th2-high asthma and Th2-low asthma in GSE41861. **(E)** Violin plots show the expression levels of CST1 in Th2-high asthma, Th2-low asthma and controls in GSE67472. **(F)** ROC curves of CST1 expression levels in bronchial epithelium discriminating Th2-high asthma and Th2-low asthma in GSE67472. **(G)** Correlation analysis and scatter plot of CST1with CLCA1 expression levels in bronchial epithelium in GSE41861. **(H)** Correlation analysis and scatter plot of CST1 with IL13 expression levels in bronchial epithelium in GSE41861. **(I)** Correlation analysis and scatter plot of CST1 with CLCA1 expression levels in nasal epithelium in GSE101720. AS, Asthma; ns, no significance; *p < 0.05.

32 bronchial epithelial samples from asthma patients were classified as Th2-high asthma group according to the expression of three signature genes (SERPINB2, POSTN, and CLCA1) of type 2 inflammation using Euclidean distance clustering analysis, and the remaining 19 samples were assigned to the Th2-low asthma group (**Figure 7A**). The expression level of CST1 in the Th2-high asthma group was significantly higher than that in the Th2-low asthma group, but there was no significant difference between the Th2-low asthma group and the control group (**Figure 7B**). The ROC curves indicated that CST1 in the bronchial epithelium exhibited excellent diagnostic efficiency for Th2-high asthma in GSE41861 dataset (**Figure 7C**). In addition, it is worth noting that the CST1 expression level in the nasal epithelium also showed an ideal diagnostic efficiency for Th2-high asthma (**Figure 7D**). Finally, we validated the expression level of CST1 in Th2-high asthma group, Th2-low asthma group, and healthy control group (**Figure 7E**), and the diagnostic efficiency of CST1 in distinguishing Th2-high asthma from Th2-low asthma by using the GSE67472 dataset (**Figure 7F**). For the Th2 cytokines, CST1 was significantly correlated with CLCA1 and IL-13 in the bronchial epithelium of asthmatic patients (**Figures 7G, H**) and CLCA1 in the nasal epithelium of AR patients (**Figure 7I**).

### 3.8 Gene set enrichment analysis

**Figure 8:**
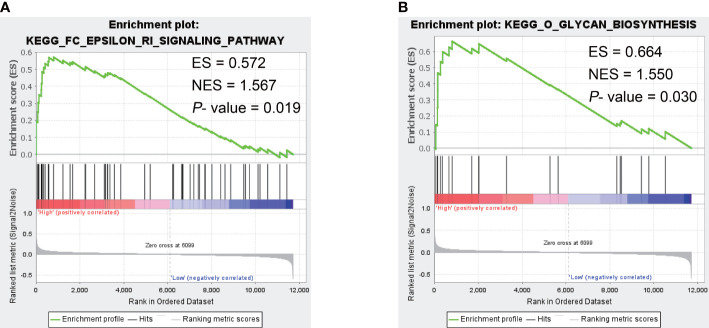
The GSEA result of CST1. **(A)** Fc epsilon RI signaling pathway. **(B)** O-glycan biosynthesis. ES, Enrichment Score; NES, Normalized Enrichment Score.

Since CST1 was the only key gene that was up-regulated in the nasal epithelium and bronchial epithelium of both AR and asthma patients, and the correlation heatmap showed that CST1 was the most correlated gene with the other three key genes, we speculated CST1 might be the primary driver among key genes and was more likely to be an effective target for drug therapy. Then we selected CST1 for GSEA analysis, which showed that the CST1 high expression group was significantly involved in the Fc epsilon RI (FcϵRI) signaling pathway (NES = 1.567, *P*-value = 0.019) (**Figure 8A**) and O-glycan biosynthesis (NES = 1.550, *P*-value = 0.030) (**Figure 8B**).

### 3.9 The mRNA-miRNA-lncRNA ceRNA network in asthma

**Figure 9:**
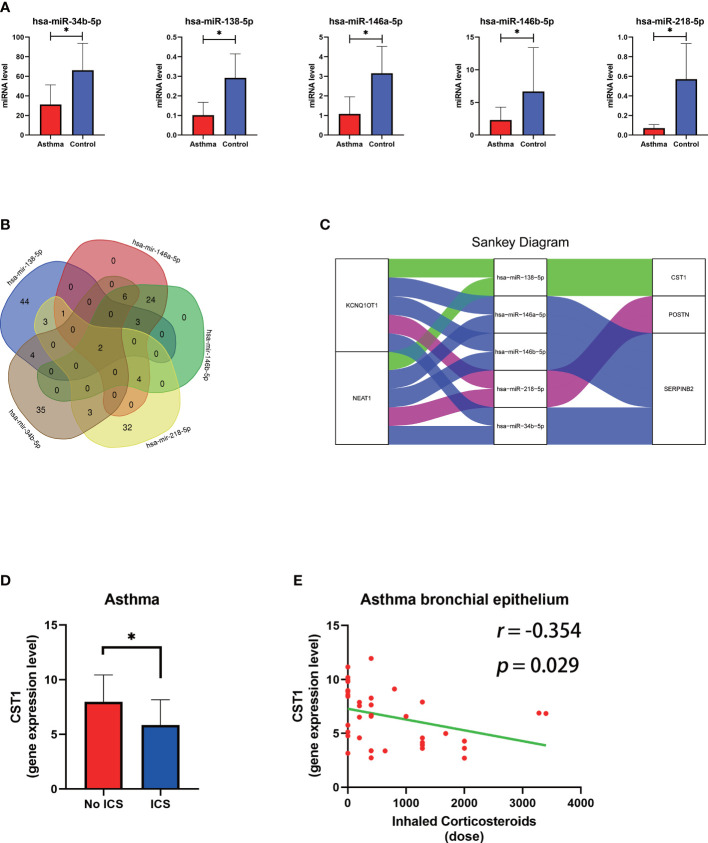
The construction of the lncRNA–miRNA–mRNA ceRNA network of key genes and relationship between ICS and CST1 expression levels. **(A)** Histogram of the expression levels of five predicted miRNAs in the bronchial epithelium of asthma patients. **(B)** Venn diagram of lncRNA predicted by the miRNet database involved in the regulation of all miRNAs. **(C)** The sankey diagram of the ceRNA network. **(D)** Histogram of CST1 expression levels in the bronchial epithelium of asthma patients with and without ICS. **(E)** Correlation analysis and scatter plot of CST1 expression level in bronchial epithelium of asthma patients and ICS dose. ICS, Inhaled Corticosteroids. P < 0.05.

We obtained 5 unique miRNAs, hsa-miR-138-5p, hsa-miR-146a-5p, hsa-miR-146b-5p, hsa-miR-34b- 5p, and hsa-miR-218-5p, by combining TargetScan, miRNet database and GSE142237 analysis results, all were down-regulated in asthma (**Figure 9A**) (**Supplementary Table S5**). The miRNet database predicted two lncRNAs (KCNQ1OT1 and NEAT1) involved in the regulation of all miRNA (**Supplementary Table S6**) (**Figure 9B**). Finally, a 10-axis ceRNA network containing 2 LncRNAs, 5 miRNAs, and 3 key genes was constructed (**Figure 9C**).

### 3.10 Small molecule drug candidates for comorbidity prevention

DEGs in bronchial epithelium of AR patients and their expression trends were imported into the CMap database to predict potential drugs which could reverse the altered expression of DEGs. Subsequently, we screened the top 3 small-molecule compounds or drugs that could reverse DEGs by enrichment score ranking, including Alfaxalone, QL-XI-92, Deferiprone. In addition, DEGs in nasal epithelium of asthma patients were uploaded to the CMap, and the results showed that verrucarin A was the drug that co-occurred and ranked highest in the results of both groups (**Table 6**). These compounds have the potential to prevent the development of asthma in AR patients. The results of the analysis of GSE89809 showed that corticosteroids could down-regulate the expression level of CST1 (**Figure 9D**) and that the dose of ICS was significantly and negatively correlated with CST1 expression levels (**Figure 9E**).

**Table 6:** Four compounds predicted using the CMap database.

GSE	Rank	Name	ID	Description	Score
101720/41861	4/16	Verrucarin-A	A26002865	Protein synthesis inhibitor	-87.1/-77.46
101720	1	Alfaxalone	K41445866	Chloride channel agonist	-93.41
101720	2	QL-XI-92	U73238814	DDR1 inhibitor	-89.58
101720	3	Deferiprone	K06878038	Chelating agent	-89.28

GSE, GEO Series.

## 4 Discussion

AR usually precedes the development of asthma, with a large proportion of children and adults developing asthma within 2 years of an AR onset (36). Bronchial hyperresponsiveness is common in AR patients even in the absence of asthma symptoms, which is also associated with an increased risk of future asthma (37). Clinically, asthma attacks often co-occur with worsening nasal symptoms, and patients with severe or poorly controlled AR have poorer asthma control compared with patients with mild or well-controlled AR (38). Effective AR treatment can effectively prevent or delay asthma attacks (39, 40). Therefore, identifying biomarkers of the association between AR and asthma is important for promoting therapeutic and preventive strategies. In this study, by applying WGCNA to analyze transcriptomic data from the upper and lower airways, we found that CST1 and its related molecular functions played a key role in AR comorbid asthma and were potential biomarkers of airway allergic diseases.

GO analysis of DEGs in the bronchial epithelium of AR patients indicated that DEGs were mainly involved in inflammatory and immune responses. Notably, in terms of molecular function, DEGs were significantly enriched on CXC chemokine receptor (CXCR) related entries. Studies have demonstrated that CXCR3 played a key role in the development of allergic airway inflammatory responses by mediating lung T cell homing (41). Another study using single-cell RNA-sequencing found that CXCR4 signaling in subsets of CD8+ T cells and monocyte clusters was associated with exacerbation in patients with asthma (42). Radermecker et al. identified locally programmed CXCR4^hi^ lung neutrophils that released neutrophil extracellular traps as an early trigger of type 2 allergic airway inflammation in a mouse model of asthma (43). In addition, KEGG analysis showed that asthma was the most significant pathway enriched for DEGs in the bronchial epithelium of AR patients. In conclusion, these results suggest that gene expression in the lower airways of AR patients has a trend toward asthma despite the absence of asthma, explaining how allergic rhinitis may play a role as a risk factor for asthma development. We also found that DEGs in the nasal epithelium of asthmatic patients were mainly involved in the regulation of cysteine-type endopeptidase activity, further indicating the importance of CST1 as a common biomarker of upper and lower airways.

CST1 encodes an endogenous cysteine protease inhibitor (44). It was reported that the expression of CST1 was increased in the nasal epithelium of patients with seasonal AR, and the addition of exogenous CST1 to basophils could inhibit Japanese cedar, Cryptomeria japonica allergen-stimulated histamine release *in vitro* (45). Another study found that downregulation of CST1 suppressed inflammatory responses and mucus production in AR (46). In addition, CST1 is also upregulated and acted as a potent upstream initiator of epithelial-derived type 2 inflammation in chronic rhinosinusitis with nasal polyps (47, 48), another nasal disease that is closely related to asthma (49). Recent studies have further shown differential expression of CST1 in airway epithelial cells in exercise-induced bronchoconstriction (50). However, unlike the above studies, Fukuoka et al. found that CST1 was able to protect the nasal tight junction barrier by inhibiting allergen protease activity in Japanese cedar induced AR mouse model, thereby suppressing AR symptoms (51), but this study has not been confirmed in human subjects. In conclusion, the above studies suggest that CST1 may be an important mediator and biomarker of allergic diseases and that it plays a key role in the pathogenic mechanisms of asthma and AR.

CST1 was the only key gene that was upregulated in both the upper and lower airways in patients with asthma and AR, and its expression levels in the upper and lower airways had a high correlation. In addition, the correlation heatmap showed that CST1 had the highest correlation coefficient sum. A recent study found that recombinant CST1 induced type 2 epithelial cytokine secretion and Th2 cell infiltration in a time-dependent manner in the nasal mucosa of healthy mice, producing marked type 2 inflammation (47). These results indicated that CST1 was in the upstream position of the regulatory network in the process of inflammation. The upregulation of CST1 in the entire airway (including nasal mucosal epithelium and bronchial epithelium) lead by AR may act as a primary driver with an initiating factor in the development of asthma in AR patients.

Our results showed that CST1 was upregulated in Th2-high asthma group compared with Th2-low asthma group, in which the Th1-asthma was included, and there was no significant difference between the Th2-low asthma group and the healthy control group. ROC curves also displayed that CST1 in nasal and bronchial epithelium both manifested excellent diagnostic efficiency for Th2-high asthma. Correlation analysis showed a strong correlation between CST1 and type 2 inflammatory signature genes, both in the nasal epithelium and bronchial epithelium. Moreover, Th2-asthma contains more eosinophils in the airway epithelium compared to non-Th2 asthma, which is predominantly neutrophilic (52, 53). This is also consistent with the results that CST1 is inversely correlated with neutrophils. These data suggest the specificity of CST1 towards Th2 responses. In addition, asthma developed from AR may have higher levels of CST1 expression and be more prone to Th2-high asthma, which is associated with high airway reversibility and more significant reductions in lung function compared to non-Th2 asthma (54, 55). This may also explain why asthma with AR is more severe and difficult to control, and treatment of AR can improve asthma symptoms and reduce the number of asthma attacks.

Analysis of CST1 expression levels in different comorbidities revealed that in the nasal epithelium of AR, CST1 expression levels were not significantly altered, regardless of whether asthma was accompanied or not and whether asthma was controlled or not. The expression of CST1 in the nasal epithelium in asthma does not appear to be additive with that in AR, suggesting that the inflammatory disease of the upper and lower airways is a whole. In the lower airways, CST1 was highly expressed in AR comorbid asthma patients compared with AR alone. Unfortunately, due to the lack of data on CST1 expression in the lower airways of patients with asthma alone on the same platform, we could not know whether the expression level of CST1 is higher in patients with asthma comorbid AR than patients with asthma alone. Subsequently, a 10-axis ceRNA network containing 2 LncRNAs, 5 miRNAs, and 3 key genes was constructed to reveal the regulatory mechanism of CST1. In the network, the lncRNAs KCNQ1OT1 and NEAT1 compete with all miRNAs and have the potential to be key targets in the treatment of AR and asthma.

Allergen-activated CD4 T cells in the airways play a key role in eosinophil recruitment, inflammatory response, and mucus production, ultimately leading to airway obstruction in asthmatics (56, 57). Equally important, CD4 memory T cells localized in lung tissue may be responsible for the persistence of the disease and the ability to trigger periodic worsening of lung inflammation upon encountering airborne allergens (58, 59). Based on our result that CST1 was significantly positively correlated with activated CD4 memory T cells, it can be inferred that blocking the expression of CST1 in the airway may prevent the development of asthma by inhibiting the activation of CD4 memory T cells. In addition, we also demonstrated a negative correlation between CST1 and M2 macrophages, which was mainly involved in anti-inflammatory responses and could be functionally reversibly re-differentiated by the local cytokine environment *in vitro* into pro-inflammatory M1 macrophages (60). Thus, CST1 may also promote airway inflammation by participating in the re-differentiation of M2 macrophages to M1 macrophages.

GSEA analysis showed that high CST1 expression group was significantly associated with the FcϵRI signaling pathway and O-glycan biosynthesis. In asthma, mast cells are activated primarily through IgE-mediated cross-linking of FcϵRI to allergens (61). With the in-depth study of allergy mechanisms, it has also been found that IgE can bind carbohydrates, and more and more scholars believe that glycans are a kind of allergens (62). The major allergen of Artemisia vulgaris, Art v 1, has been shown to be O-glycosylated in two ways, and IgE was found to bind to the β-arabinosyl residues of Art v 1 (63). Another study demonstrated that IgE could bind to O-glycans of potato lectin (64). It is not clear whether CST1 interacts with FcϵRI and O-glycan. However, a previous study found a significant increase in the number of intraepithelial mast cells in Th2-high asthmatics compared with Th2-low asthmatics (65), and our results showed CST1 was highly expressed in Th2-high asthma. It can be speculated that the high expression of CST1 may be involved in IgE-mediated activation of mast cells through glycosylation of allergens and cross-linking with FcϵRI, thus leading to the inflammatory response of airway allergic diseases.

Analysis of the CMap database identified verrucarin A was the drug that co-occurred and ranked highest in the results of both groups, which could inhibit protein biosynthesis by interfering with the activity of peptidyl transferase, and regulate many signaling pathways that promote apoptosis, but its potent toxicity to normal non-dividing tissue has prevented its use in humans (66). By binding to small-molecule ligands, verrucarin A is able to act centrally in target cells, minimizing toxicity to non-targeted tissues. A recent study found that a folate-verrucarin A conjugate can successfully treat inflammatory peritonitis in mice by depleting inflammatory macrophages without killing other cells in the peritonitis fluid (67), indicating that verrucarin A deserves further exploration as a potential therapy for preventing the development of asthma in AR patients. Previous studies also confirmed that glucocorticoid treatment of upper airway inflammation could indirectly improve asthma symptoms and reduce bronchial hyperresponsiveness (34). Our study found that corticosteroids could down-regulate the expression level of CST1, and the expression levels of CST1 in the upper and lower airways were positively correlated, suggesting that CST1 played an important role in airway allergic diseases, and targeted therapy against CST1 might be important in preventing asthma development in AR patients.

Our study also has some limitations. We used data from public databases for our analysis, which were from different platforms and could not be compared directly. There were certain differences in the inclusion criteria of the datasets, a general lack of corresponding clinical data, and a small clinical sample in some datasets. In addition, our study is limited to the transcriptome level, and the significance of the findings requires further validation through prospective clinical and basic experiments.

In conclusion, our study identified CST1 as a potential biomarker for airway allergic diseases by applying WGCNA, analyzing the upper and lower airway transcriptomic data of AR and asthma. It provides a new perspective on the pathogenesis of AR comorbid asthma and provides new research clues for preventing the development of asthma in AR patients.

## Data availability statement

The datasets presented in this study can be found in online repositories. The names of the repository/repositories and accession number(s) can be found in the article/**Supplementary Material**.

## Author contributions

XL, XF and MW designed the study. MW drafted the manuscript. MW, LG, YL, SH, XZ, and XX performed the analysis. XF and XL contributed to interpretation of the results, reviewed and edited the manuscript. All authors contributed to the article and approved the submitted version.
