# Supplementary material for: Transcriptomic analysis of asthma and allergic rhinitis reveals CST1 as a biomarker of unified airways
Source: Front Immunol. 2023 Jan 17;14:1048195. doi: 10.3389/fimmu.2023.1048195 (PMC9888248; doi:10.3389/fimmu.2023.1048195)
Supplement: Supplementary file 1 [file Table_1.docx]

**Supplementary Table S1.**

**Detailed information on the inclusion criteria and diagnostic criteria for each dataset.**

| **GSE** | **Detailed information** |
| --- | --- |
| **GSE19187** | The dataset contained gene expression profiles of nasal epithelial cells from 14 children with AR and, 6 children with AR and concomitant uncontrolled asthma, 7 children with AR and concomitant controlled asthma, and 11 healthy controls. The diagnosis of AR was based on clinical criteria (Allergic Rhinitis and its Impact on Asthma (ARIA) guidelines：rhinorrhea, nasal congestion, itching, and sneezing) and a positive pinprick test. Asthma was defined by the following clinical criteria (Expert Panel Report 3 (EPR3) 2007): recurring episodes of wheezing, dyspnea, cough, and obstructive syndrome reversible under beta2-mimetic. All children presented with mild or moderate to severe perennial AR. All had not received nasal corticosteroids therapy for at least 1 month at the time of recruitment. Healthy control children were selected on the basis of the absence of any personal or family history of allergic disease. Prior to enrollment, all subjects were clinically examined to exclude those with symptoms of infectious rhinitis or bronchitis within the past 15 days. Nasal epithelial sampling was performed by applying a cytological brush to the inferior turbinate and adjacent nasal mucosa. |
| **GSE67472** | The dataset contains gene expression profile data from bronchial epithelial brushes obtained from 62 subjects with mild to moderate asthma (Th2-high = 40, Th2-low = 22) without inhaled steroid use and 43 control subjects without asthma. |
| **GSE41861** | The dataset contains gene expression profiling data from nasal epithelial and bronchial epithelial brushes from 54 asthma patients and 30 healthy control subjects, all samples from Nutley Translational Research Sciences Hoffmann-LaRoche, New Jersey, USA. |
| **GSE101720** | The dataset containing gene expression profiling data from nasal and bronchial epithelial brushes of 7 asthma comorbid AR, 10 AR and 9 healthy subjects. The AR diagnosis was based on the ARIA 2010(25),and asthma diagnosis was based on the 2012 Global Initiative for Asthma and EPR3 2007 . |
| **GSE89809** | The dataset containing bronchial epithelial gene expression profile data from 38 asthma patients, 13 of whom were not on inhaled corticosteroids medication and the remaining 25 patients inhaled different doses of corticosteroids for asthma. |
| **GSE142237** | The dataset containing bronchial epithelial microRNA expression profiles obtained by bronchoscopy from 8 asthma patients and 4 healthy controls. |

Note: All datasets are in compliance with GEO's upload regulations, and have been background corrected and normalized
