# Supplementary material for: Transcriptomic analysis of asthma and allergic rhinitis reveals CST1 as a biomarker of unified airways
Source: Front Immunol. 2023 Jan 17;14:1048195. doi: 10.3389/fimmu.2023.1048195 (PMC9888248; doi:10.3389/fimmu.2023.1048195)
Supplement: Supplementary file 4 [file Table_4.docx]

**Supplementary Table S4** The DEGs of GSE101720 and GSE41861.

| GSE | DEGs | Gene names |
| --- | --- | --- |
| GSE101720  Nasal epithelial cells | Up-regulated | DDX3Y TXLNG2P UTY USP9Y RPS4Y1 KDM5D ZFY TTTY15 EIF1AY LINC00278 PRKY NLGN4Y CST1 TBL1Y TMSB4Y FAM8A4P TTTY14 ARSEP1 FETUB KALP RP11-424G14.1 TREM1 CLCA1 SLC26A4 GUCY1B2 FPR2 SLC9A3 IL1RL1 OSM HK3 IDO1 CAPN14 UGT2B7 AQP9 FCGR3B SNTG2 HLA-DQB1 TRIM31 APOBEC3A MIR3648 OASL RP11-44K6.2 SLC26A4-AS1 AL161626.1 SH2D1B EMR1 MIR3687 CD274 RP11-32B5.1 ARHGAP40 SERPINB4 RN7SL471P HLA-V HMCN2 SYT5 AL592188.4 SECTM1 DPP4 AC144450.2 TNNI3 RNA18S5 PAX8- S1 SERPINB2 RNA28S5 GCNT3 SLCO4A1 RP11-706O15.3 HLA-DQA1 SCUBE1 DOC2B AC017060.1 CCL4 PLAUR MT-ND3 LINC00930 IL8 DQX1 SRGN MT- TP8 AL592188.2 AL592188.1 MYO7A WARS SOCS2 AC026740.1 SMIM6 SEMA7A ALOX5AP PLEKHN1 GSDMC AC011043.1 ANKRD36C RP5-884M6.1 TAP1 CTD-2228K2.5 FAM153B ADAMTSL4 HAS3 RGS2 DNAJC12 NLRC5 MUC4 TNFSF13B GPX1P1 GPR65 MT-TI AC009133.14 MTATP6P1 P2RY6 AC009133.12 SAMD9L ANKRD20A5P ASMTL-AS1 OIT3 HLA-DRB1 MLKL RP11-434I12.3 |
|  | Down-regulated | AMOTL+R3:R1011 CORO2B NRCAM GSTM3 PKIA MAOB SORCS2 PTRF CNTN1 CXorf57 C16orf89 FAM171B CRABP2 SIAH3 LRRC37A6P SNCG KBTBD11 PROS1 ANKH ZFR2 PEG10 EPDR1 SNAI2 COL6A3 MGP STATH CDH7 DPP10 NPAS3 MARVELD1 KAL1 DOK6 RP11-627G23.1 PTPRD SCN4B COL17A1 COL12A1 PEX5L GJA1 TSPAN11 COL14A1 RP11-742B18.1 SLITRK5 FMOD B3GALT5 KCNJ5 SLC47A1 FBN1 TGFB2 RP11-3N2.13 CYP2A6 ISLR CDH13 ZNF667-AS1 TENM2 NLRP2 DDIT4L C21orf88 SLC6A15 LPHN3 DLK2 RP11-1220K2.2 COL19A1 PPP1R1B CNTN4 SLC16A12 NDNF PNMAL1 PAMR1 CDH11 ST6GAL2 COL1A2 SCGB1A1 COL3A1 HLA-DQA2 SPARC PAPPA JAM3 CCDC3 PREX2 MMP2 SPOCK3 DKK3 FDCSP RP11-687D19.1 RUNX1T1 BPIFB2 GFRA1 CTD-2626G11.2 IGJ DCN CYS1 LTF IGHA1 FN1 SCN2B HP DMBT1 CD200R1L |
| GSE101720  Bronchial epithelia cells | Up-regulated | DDX3Y USP9Y UTY TXLNG2P KDM5D RPS4Y1 ZFY TTTY15 PRKY EIF1AY FAM8A4P ARSEP1 LINC00278 TTTY14 NLGN4Y CST1 TMSB4Y RP11-424G14.1 KALP CPA3 FETUB TPSAB1 MS4A2 PTCHD4 IL1RL1 SLC18A2 GATA2 GUCY1B2 POSTN TPSB2 ZMAT4 TFF1 HLA-DQB1 NRAP CEACAM21 CEACAM5 CXCL11 FAM177B MUC5AC USP32P1 AC011043.1 SH2D1B GNLY PAX8-AS1 RP11-44K6.2 RP11-32B5.1 CXCL9 LINC00920 TESPA1 |
|  | Down-regulated | PSCA CTC-550B14.7 ESPNP CILP RP11-379F4.7 LYPD2 PAPPA TMPRSS11A NLRP2 RP11-815M8.1 SELP DDIT4L CSF3 H19 RP11-384F7.2 HLA-DQA2 B3GALT5 C21orf88 XIST |
| GSE41861  Bronchial epithelia cells | Up-regulated | CLCA1 CPA3 CST1 SERPINB2 PRR4 POSTN SERPINB10 RGS13 MS4A2 SIGLEC6 ITLN1 |
|  | Down-regulated | FOS KCNA1 LOC285419 FHOD3 C3 PNMA2 SCGB3A1 MUC5B C6 GRP TMEM45A WIF1 BPIFA1 |
| GSE41861  Nasal epithelia cells | Up-regulated | CPA3 CST1 POSTN RGS13 CCL26 ITLN1 SAMSN1 CST4 CLCA1 MS4A2 CD200R1 CSF2RB HPGDS PP7080 SERPINB10 SIGLEC6 |
|  | Down-regulated | MUC2 |

GSE, GEO Series; DEGs, differentially-expressed genes.
